# Integrative morphometric and molecular analyses reveal possible genetic contamination of silver catfish populations of the genus *Rhamdia* in Neotropical River basins

**DOI:** 10.1111/jfb.70057

**Published:** 2025-04-16

**Authors:** Thaís Souto Bignotto, Herivelto Beck de Souza, Rafael Clovis da Silva Bronzim, Thiago Cintra Maniglia, Dirceu Baumgartner

**Affiliations:** ^1^ Centro de Engenharias e Ciências Exatas Universidade Estadual do Oeste do Paraná (Unioeste) Paraná Brazil; ^2^ Programa de Pós‐Graduação em Ciências Ambientais Unioeste Paraná Brazil; ^3^ Programa de Pós‐Graduação em Recursos Pesqueiros e Limnologia Unioeste Paraná Brazil; ^4^ Coordenação de Engenharia de Bioprocessos e Biotecnologia Universidade Tecnológica Federal do Paraná (UTFPR) Paraná Brazil

**Keywords:** aquaculture, barcodes DNA, fish farm, interspecific hybrids, Jundiá, molecular markers

## Abstract

*Rhamdia quelen*, *Rhamdia branneri* and *Rhamdia voulezi* are morphologically similar species that, until recently, were considered synonymous. Although *R. quelen* has wide distribution in the Neotropical region, *R. branneri* and *R. voulezi* are sympatric and endemic species of the Iguaçu River basin. We used an integrative approach, including morphometric and molecular data (barcodes DNA, *COI* gene), to assist in the identification and delimitation of these species. We also intended to investigate genetic contamination of the Paraná III and lower Iguaçu River basins, as silver catfish production has increased in southern Brazil, and accidental or occasional escapes to nature may pose risks to the genetic integrity of native populations. *COI* sequences and morphometric data were efficient in the characterization and differentiation of *Rhamdia* species and may be helpful tools in correctly identifying *R. quelen*, *R. branneri* and *R. voulezi*. Both morphometric and molecular analyses indicated the segregation of specimens into three groups. Although this separation coincided with the taxonomy and the collection site in the morphometric analyses, the taxonomic identification of most samples did not coincide with the molecular identification. This fact may be due to (i) incorrect morphological identification and/or (ii) escapes of pure species and/or interspecific hybrids from fish farms. The detection of *COI* haplotypes of *R. quelen* in the lower Iguaçu River, as well as *COI* haplotypes of *R. branneri* and *R. voulezi* in the Paraná III basin, combined with the morphometric and morphological characteristics of the specimens, reinforces the occurrence of hybrid specimens in these river basins. These results reveal the importance of characterizing species and interspecific hybrids of *Rhamdia* and the urgency to regulate aquaculture activities.

## 1 INTRODUCTION

Fish of the genus *Rhamdia* (Siluriformes, Heptapteridae) are endemic to the Neotropical region, occurring from Argentina to Mexico (Bockmann & Guazzelli, 2003). Within the family Heptapteridae, the genus *Rhamdia* was considered the most specious, with approximately 100 nominal species described. However, in a taxonomic review, Silfvergrip (1996) reduced the number of previously described species to only 11. Their morphological and meristic analyses identified 47 nominal species as synonymous of *Rhamdia quelen* (Silfvergrip, 1996). However, some authors (e.g., Angrizani & Malabarba, 2020; Hernández et al., 2015; Perdices et al., 2002) contest this review, highlighting that the analyses conducted by Silfvergrip (1996) present some gaps in the geographical distribution of the examined specimens and lack robust characters for the diagnosis of *R. quelen* (Angrizani & Malabarba, 2020). The proposed diagnosis for the species includes several different morphotypes under a single name, making *R. quelen*, sensu Silfvergrip (1996), a complex of several species (Angrizani & Malabarba, 2020).

Among the synonymous species of *R. quelen* (Quoy & Gaimard 1824), sensu Silfvergrip (1996), are *Rhamdia branneri* Haseman 1911 and *Rhamdia voulezi* Haseman 1911, both described as endemic to the Iguaçu River basin (Baumgartner et al., 2012). Nonetheless, studies have supported the validity of the three species by identifying morphological, morphometric, ecological, cytogenetic and molecular differences among them. Although *R. branneri* and *R. voulezi* have the same diploid number and karyotype formula, karyotypic differences in the number and type of B chromosomes were identified (Abucarma & Martins‐Santos, 2001). Besides that, *R. branneri* and *R. voulezi* were considered valid species not only due to the morphological and karyotypic divergences between them but also because of the ichthyofaunistic endemism of the Iguaçu River basin (Baumgartner et al., 2012). Likewise, ecomorphological and morphometric characteristics were used for the reappraisal of *R. branneri* and *R. voulezi*, affirming that they are valid species (Garavello & Shibatta, 2016; Mise et al., 2013). Finally, studies based on nucleotide sequences of the mitochondrial gene cytochrome c oxidase I (*COI*; barcodes DNA), identified three molecular operational taxonomic units (MOTUs) among the analysed specimens, evidencing the validity of *R. quelen*, *R. voulezi* and *R. branneri* (Ribolli et al., 2017; Scaranto et al., 2018).

In southern Brazil, fish's tolerance to low temperatures enables the production of *Rhamdia* species. However, because the identification and separation of morphologically similar species are difficult, and the cultivation of *R. quelen, R. voulezi* and *R. branneri* is deliberately performed using the same common name ‘jundiá’, interspecific crosses and contamination of wild stocks can occur (Hashimoto et al., 2014; Scaranto et al., 2018).

Integrative analyses addressing traditional and molecular methods in taxonomic identification can promote an agile and accurate species identification (Ribolli et al., 2017; Rocha et al., 2022; Steinke & Hanner, 2011), avoiding problems with the breeding of the species. Morphometric analyses have been used to reveal differences in body shape relative to fish size (Almeida et al., 2012; Garavello & Shibatta, 2016), which can help elucidate systematic relationships and provide better interpretation and comparison of quantitative character variation patterns (Blackith & Reyment, 1971; Rocha et al., 2022). Molecular methods can aid the delimitation and identification of species. A partial region of the mitochondrial *COI* gene has been employed in identifying animal species, as proposed by Hebert et al. (2003, 2004, barcodes DNA). However, success in identifying species from molecular data depends on accurate taxonomic identification and the availability of reference nucleotide sequences (Ribolli et al., 2017; Ward et al., 2009). In addition, the delimitation of cryptic species can be resolved with relevant MOTUs, complementing the limitations of the traditional methods (Blaxter, 2006; Scaranto et al., 2018).

Given the morphological complexity of *Rhamdia* species and the taxonomic evidence found in previous studies, the present study sought to characterize the silver catfishes *R. quelen*, from the Paraná III River basin, and *R. branneri* and *R. voulezi*, from the lower Iguaçu River basin, with morphometric and molecular analyses, to assist in the identification and delimitation of the species occurring in these basins. Also, it is intended to investigate the possible genetic contamination of rivers in the Paraná III and lower Iguaçu basins.

## 2 MATERIALS AND METHODS

### 2.1 Ethics statement

The specimens were collected under the authorization of Instituto Chico Mendes de Conservação da Biodiversidade (ICMBio), permit number 19026–7. This study was carried out under the approval of the Ethics Committee on the Use of Animals of Universidade Estadual do Oeste do Paraná (Experimental Certificate in the Use of Animals in Research 13/24 – CEUA/Unioeste).

### 2.2 Biological material and sampling

**FIGURE 1:**
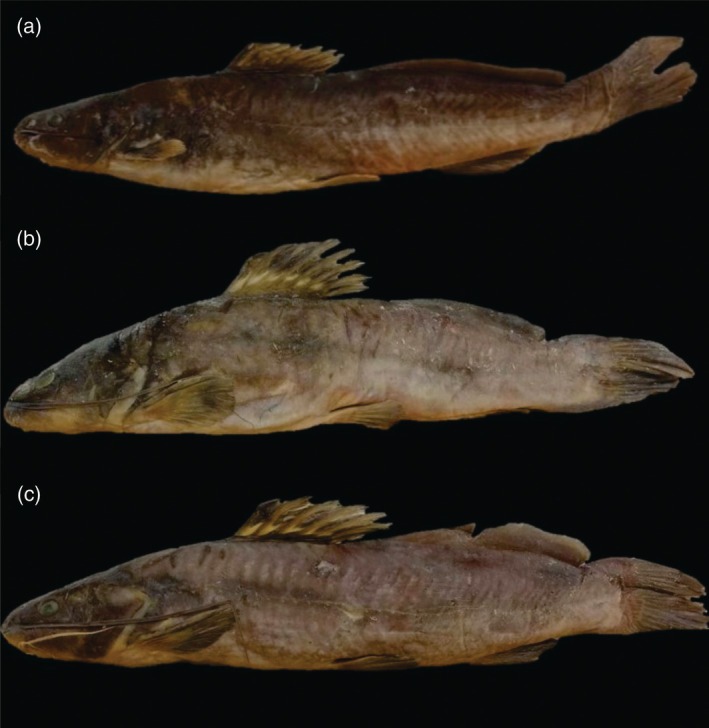
Specimens of (a) *Rhamdia quelen*, of the Paraná III River basin, (b) *Rhamdia branneri* and (c) *Rhamdia voulezi*, endemic to the lower Iguaçu River basin, Paraná, Brazil. (*Source*: Souza, 2017).

**FIGURE 2:**
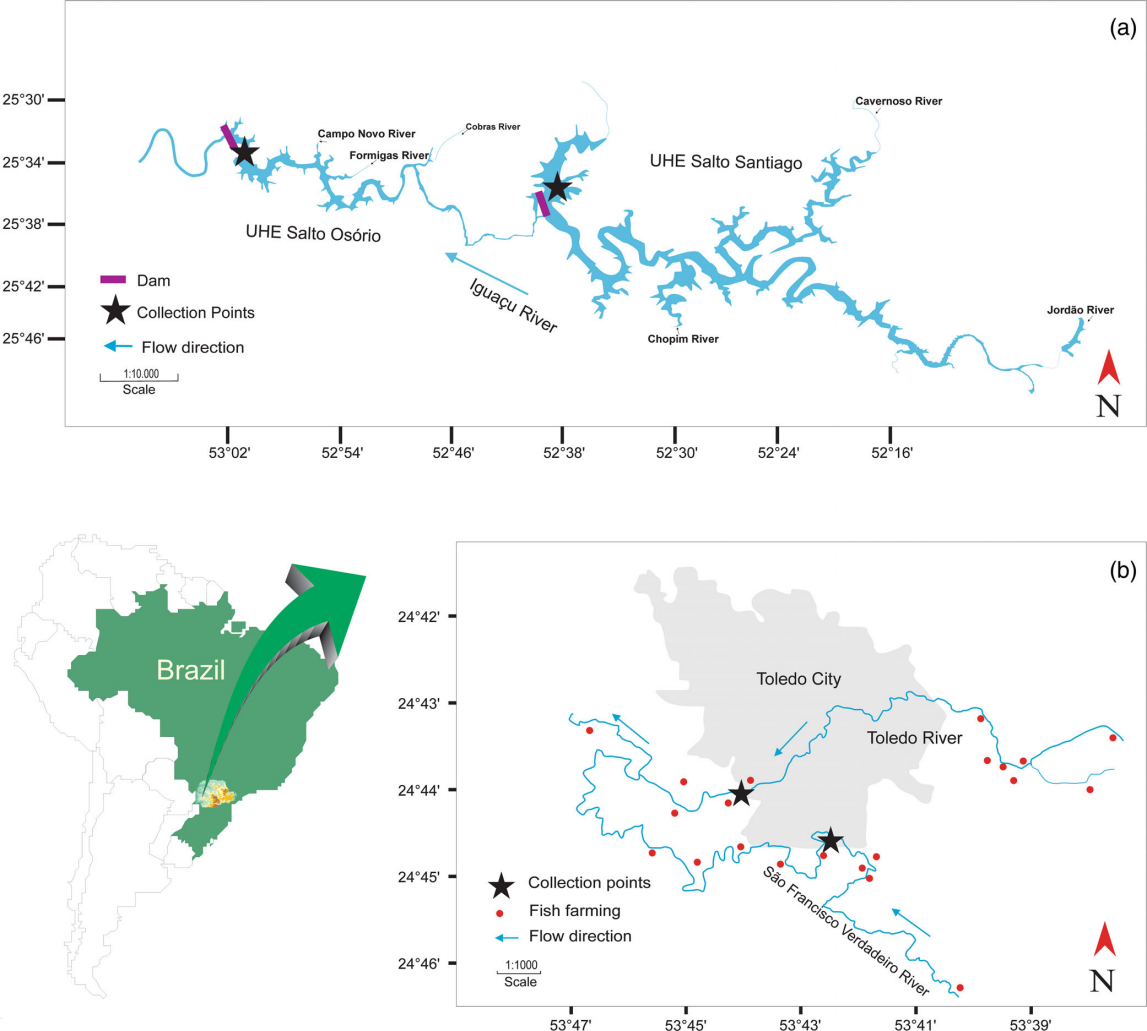
Study area in (a) lower Iguaçu and (b) Paraná III river basins.

Thirty specimens of *R. quelen* (Figure 1) were sampled in the Paraná III hydrographic basin: São Francisco Verdadeiro River (24°46′40″S, 53°43′14″W) and Toledo River (24°45′11″S, 53°45′05″W) (Figure 2). Additionally, 30 specimens of *R. voulezi* and 16 of *R. branneri* (Figure 1) were collected in two artificial reservoirs of the lower Iguaçu River: Salto Osório HPP (25°31′57″S, 52°59′30″W) and Salto Santiago HPP (25°38′24″S, 52°35′30″W) (Figure 2). Specimens from the Paraná III and the lower Iguaçu River basins were morphologically identified according to Garavello and Shibatta (2016) and Baumgartner et al. (2012), respectively. Samples were taken randomly, using fishing gear such as longlines with 2/0 and 5/0 hooks, simple waiting nets of 5, 6, 7, 8, 9, 10 and 12 cm, and trammel waiting nets of 6, 7 and 8 cm between non‐adjacent nodes. Samplings were conducted between 2015 and 2016.

After euthanasia, a muscle tissue sample was taken from each specimen and preserved in commercial ethyl alcohol in individual vials for genetic analyses. The specimens were fixed in 10% formaldehyde and maintained at the Ichthyology Laboratory of Gerpel (Research Group on Fishery Resources and Limnology) at Unioeste for morphometric analyses.

### 2.3 Morphometric analyses

**FIGURE 3:**
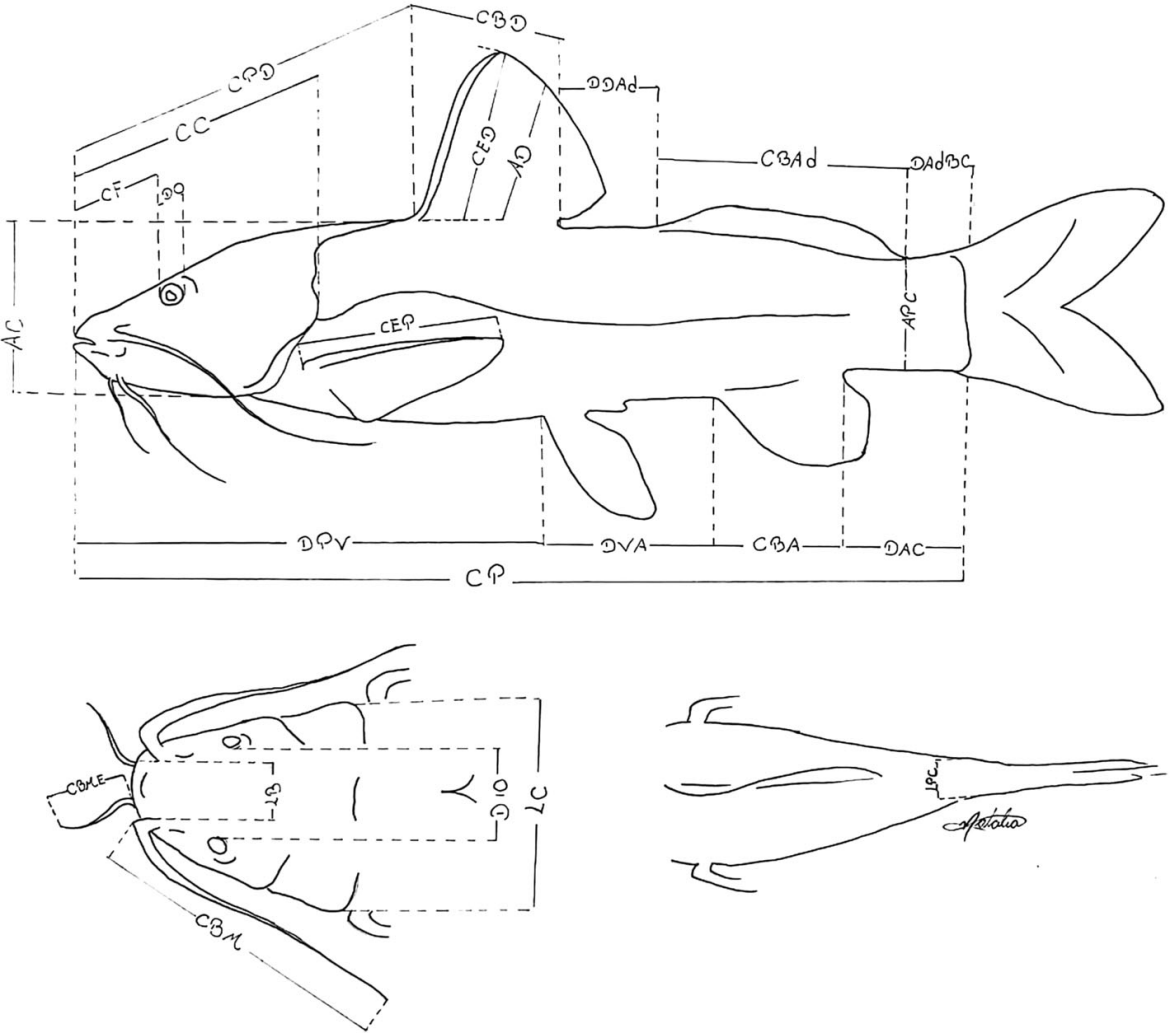
Morphometric measurements obtained from *Rhamdia* specimens of the Paraná III and lower Iguaçu river basins. See Materials and Methods for an explanation of abbreviations (adapted from Garavello & Shibatta, 2016).

Morphometric character measurements of 30 specimens of *R. quelen*, 30 of *R. voulezi* and 16 of *R. branneri* were performed using a calliper with 0.01‐mm accuracy. Morphometric measurements were obtained, according to Baumgartner et al. (2012) and Garavello and Shibatta (2016), as follows: standard length (CP), pre‐dorsal length (CPD), dorsal‐fin base length (CBD), dorsal‐fin to adipose‐fin distance (DDAd), adipose‐fin base length (CBAd), adipose‐fin to caudal‐fin base distance (DAdBC), pre‐pelvic length (DPV), pelvic‐fin to anal‐fin distance (DVA), anal‐fin base length (CBA), anal‐fin to caudal‐fin distance (DAC), caudal peduncle depth (APC), caudal peduncle width (LPC), pectoral‐fin spine length (CEP), dorsal‐fin spine length (CED), dorsal‐fin height (AD), maxillary barbel length (CBM), external mental barbel length (CBMe), head length (CC), snout length (CF), eye diameter (DO), interorbital distance (DIO), mouth width (LB), body width (LC) and body height (AC) (Figure 3).

Body proportions were made for each character relative to the standard length, except for the head subunits taken relative to the head length. Patterns of size and shape variations among morphometric variables were assessed using a multivariate statistical procedure, allowing us to verify whether the independent variable belongs to a certain group and to identify which variables influenced species segregation. For this purpose, we used discriminant analysis, a statistical technique used to classify a set of observations into distinct groups based on independent variables (Hair et al., 2005). The measurement values were transformed into variable logarithms of allometric relationships. The principal component scores for the axes of the discriminant analysis were plotted to assess size patterns and variation in morphometric data within and across populations of *R. quelen*, *R. branneri* and *R. voulezi*. Statistical analyses were performed using the Statistica 7.1 software.

### 2.4 Molecular analyses

Genomic DNA from 33 *Rhamdia* specimens (9 specimens morphologically identified as *R. quelen*, 11 as *R. voulezi* and 13 as *R. branneri*) was isolated from muscle tissues using *Wizard Genomic DNA Purification* kit (Promega) according to the manufacturer's instructions. DNA quantification was performed by visual comparison with Ladder DNA 100 base pairs (bp) on a 1% agarose gel stained with SYBR Safe. A portion of the mitochondrial gene *COI* of approximately 750 bp was amplified via polymerase chain reaction (PCR) with the primers Fish‐F1 (5′ – TCA ACC AAC CAC AAA GAC ATT GGC AC – 3′) and Fish‐R1 (5′ – TAG ACT TCT GGG TGG CCA AAG AAT CA – 3′) (Ward et al., 2005).

*COI* fragments were amplified in PCR‐independent reactions. The amplification reaction mixture consisted of 1× buffer (20 mM Tris–HCl pH 8.4 and 50 mM KCl), 1.5 mM MgCl_2_, 2.5 μM each primer, 0.4 μM each dNTP, 3 U/reaction *Taq* DNA polymerase (Invitrogen), 50 ng genomic DNA and ultrapure water to complete final 25 μL. Mitochondrial *COI* gene amplifications were performed in an automatic thermocycler programmed with the following thermal profile: an initial cycle of 4 min at 94°C, followed by 40 cycles of 45 s at 94°C, 30 s at 50–60°C and 2 min at 72°C, with a last cycle of 7 min at 72°C. The amplification efficiency was confirmed on 1% agarose gel, and the PCR product sizes were determined by comparing with the molecular size standard Ladder 100 bp.

PCR products were purified and sequenced by the company ACTGene. *COI* fragments were unidirectionally sequenced with the primer Fish‐F1. Besides, *COI* sequences from *R. quelen*, *R. branneri* and *R. voulezi*, available in the Barcode of Life Data System (BOLD; www.boldsystems.org/), derived from the study by Ribolli et al. (2017), were included in the analyses for comparisons. *Rhamdia guatemalensis* (BOLD: HBGM270‐14) was used as an out‐group. All nucleotide sequences derived from this study were deposited at GenBank (accession numbers: OR381530‐OR381565).

### 2.5 Nucleotide sequence analyses

Nucleotide sequences were edited in Bioedit Sequence Alignment Editor 7.0.1 (Hall, 1999) and aligned in Clustal Omega (Sievers et al., 2011). The identification of diagnostic and/or polymorphic nucleotides was performed visually using the Mega 11 software (Tamura et al., 2021), according to Wong et al. (2009), by highlighting variable and parsimony‐informative sites (Pi).

Phylogenetic analyses of *COI* sequences were based on the maximum likelihood (ML) and Bayesian (BA) approaches. The selection of the best evolutionary model to fit the data and the partitions of the nucleotide sequences was conducted using the PartitionFinder 2.1 software (Lanfear et al., 2012). The ML tree was constructed using the raxmlGUI software (Silvestro & Michalak, 2012), based on the partitions obtained from PartitionFinder (first, second and third bases) and the GTR + G model. The rapid bootstrap algorithm and the autoMRE function for resampling were also implemented.

The reconstruction of the BA ultrametric tree was performed using the *strict clock* molecular clock model, using the BEAST 1.8.2 software, from the input file generated by the BEAUti 1.8.0 programme (Drummond et al., 2012). The *birth–death* speciation process, which assumes constant rates of birth and death over time (or constant rates of speciation and extinction), was applied as a tree prior. Sequences were partitioned based on codon bases (first, second and third) using the HKY nucleotide substitution model. The analyses were run for 10 million generations, with a sampling frequency of 1000. The final tree was calculated after 20% burn‐in. The extent of burn‐in was determined by assessing the traits using the Tracer 1.6 software (Rambaut et al., 2014), considering an appropriate effective sample size (ESS) >200. The tree node supports were determined using posterior probabilities (PP), calculated with BEAST 1.8.2.

Genetic distance values based on *COI* sequences were estimated using Mega 11 software, based on the clusters obtained in ML and BA dendrograms and employing the K2P model. Nucleotide frequencies were obtained using Mega 11.

Three species delimitation methods were implemented to identify MOTUs (Hebert et al., 2003) and to recognize specific boundaries among *Rhamdia* specimens: (i) the Poisson Tree Process (PPS) model (Zhang et al., 2013); (ii) the General Mixed Yule Coalescent (GMYC) method, with simple boundary algorithm (Pons et al., 2006); and (iii) the Assemble Species by Automatic Partitioning (ASAP) method (Puillandre et al., 2021). Although both PTP and GMYC methods utilize an input phylogenetic tree, from which speciation fit and coalescence processes are modelled to delineate MOTUs (Larson et al., 2016; Tang et al., 2014), the ASAP procedure partition species is based on paired genetic distances. The delimitation test based on the PTP method was performed on the online server http://species.h-its.org using the ML tree that had been previously constructed. The GMYC method was implemented using the BA ultrametric tree, using the R Studio software (R Development Core Team, 2020) and the splits package (Fujisawa & Barraclough, 2013). The ASAP method was conducted on the online server https://bioinfo.mnhn.fr/abi/public/asap/, using the Kimura K80 model.

## 3 RESULTS

### 3.1 Morphometry

**FIGURE 4:**
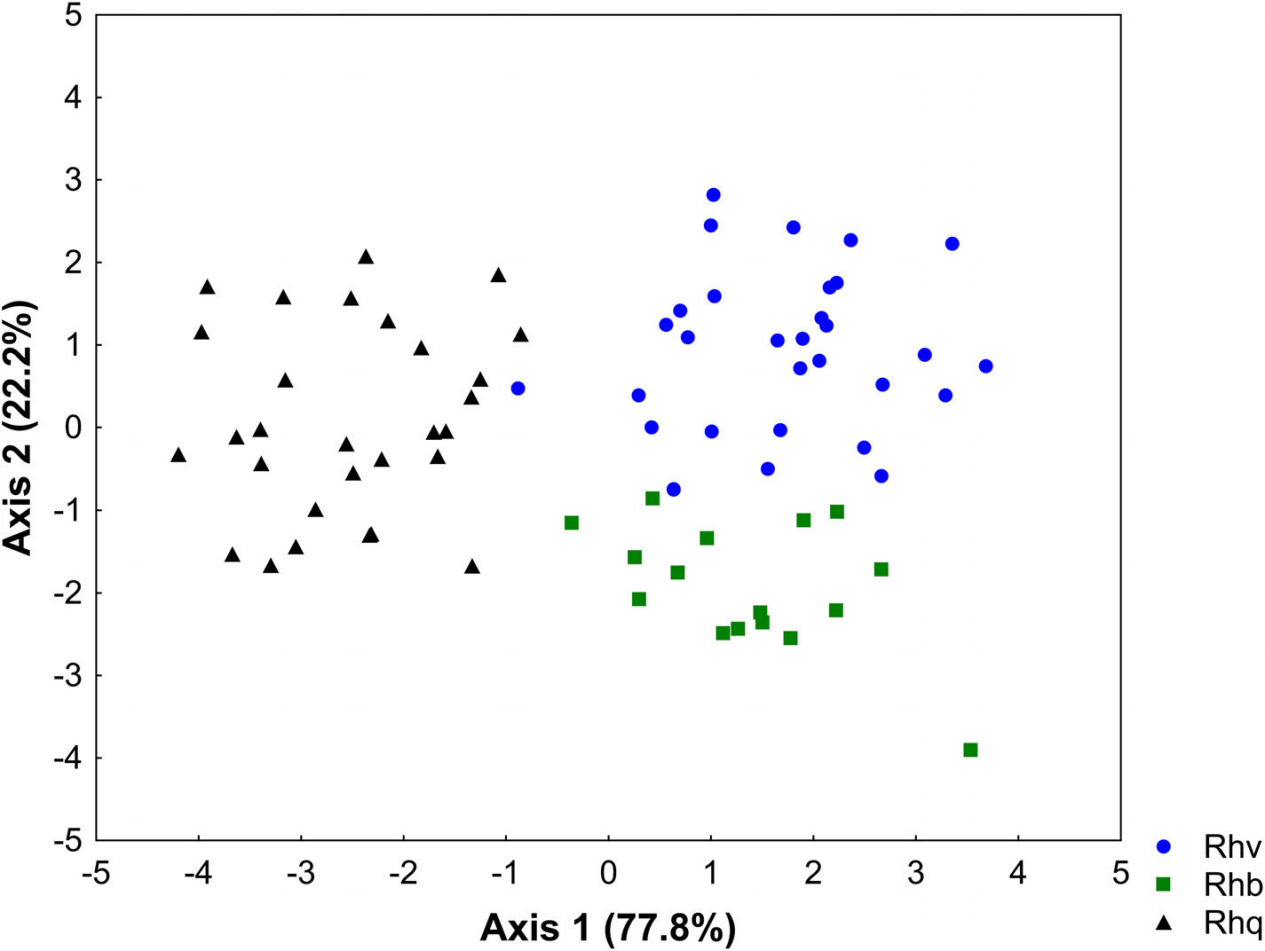
Discriminant analysis of the morphometric characters of specimens of *Rhamdia quelen* (Rhq, circles, *n* = 30) from the Paraná III River basin and *Rhamdia voulezi* (Rhv, triangles, *n* = 30) and *Rhamdia branneri* (Rhb, squares, *n* = 16) from the lower Iguaçu River basin.

The analysis assessed two discriminating axes regarding the morphometric variables of the *Rhamdia* specimens of the Paraná III and lower Iguaçu river basins (Figure 4). In general, the morphological identification coincided with the results of the morphometric analysis. There was segregation of *R. quelen* from *R. voulezi* and *R. branneri* in the first axis (77.8%). In contrast, the second axis (22.2%) indicated segregation between populations of the lower Iguaçu River, *R. voulezi* and *R. branneri*. The significant morphometric characters (*p* < 0.05) that contributed to the separation of the species were as follows: for *R. quelen*, adipose‐fin base length (CBAd), dorsal‐fin spine length (CED), eye diameter (DO) and external mental barbel length (CBMe); for *R. voulezi*, adipose‐fin base length (CBAd), dorsal‐fin spine length (CED), dorsal‐fin height (AD), snout length (CF) and maxillary barbel length (CBM); and for *R. branneri*, caudal peduncle width (LPC) and eye diameter (DO) (Table 1).

**TABLE 1:** Main components observed among *Rhamdia quelen* from the Paraná III River basin and *Rhamdia voulezi* and *Rhamdia branneri* from the lower Iguaçu River basin.

Variable	*p*‐Value	Axis 1	Axis 2
Pre‐dorsal length (CPD)	0.6134	0.0077	0.3494
Dorsal‐fin base length (CBD)	0.3900	−0.0629	0.2924
Dorsal‐fin to adipose‐fin distance (DDAd)	0.1990	0.0129	−0.4352
Adipose‐fin base length (CBAd)	**0.0001**	**−0.7130**	**0.3942**
Adipose‐fin to caudal‐fin base distance (DAdBC)	0.7779	0.0332	0.2053
Pre‐pelvic length (DPV)	0.8681	−0.0561	0.1323
Pelvic‐fin to anal‐fin distance (DVA)	0.8469	0.0969	0.1258
Anal‐fin base length (CBA)	0.8629	−0.0944	−0.0650
Anal‐fin to caudal‐fin distance (DAC)	0.5935	0.1643	−0.1893
Caudal peduncle depth (APC)	0.5358	0.2039	0.1111
Caudal peduncle width (LPC)	**0.0337**	**−0.0215**	**−4.4346**
Pectoral‐fin spine length (CEP)	0.1570	−0.0624	0.4948
Dorsal‐fin spine length (CED)	**0.0363**	**−0.5241**	**0.0179**
Dorsal‐fin height (AD)	**0.0254**	**3.7610**	**11.4780**
Maxillary barbel length (CBM)	**0.0001**	**1.4390**	**0.5099**
External mental barbel length (CBMe)	**0.0349**	**−0.5275**	**−0.8030**
Internal mental barbel length (CBMi)	**0.0201**	**−0.4398**	**0.8569**
Snout length (CF)	**0.0307**	**0.2732**	**2.8076**
Eye diameter (DO)	**0.0289**	**−4.0119**	**−11.3108**
Interorbital distance (DIO)	0.2704	0.3366	0.0662
Mouth width (LB)	0.4901	0.0046	−0.2908
Mouth height (AB)	**0.0017**	**0.5852**	**0.3830**
Body width (LC)	0.3799	0.2792	−0.1270
Body height (AC)	0.1972	−0.3880	0.0331
Eigenvalue		4.2125	1.2017
Proportional length		0.7781	1.0000

*Note*: Significant variables with *p*‐value <0.05 are presented in bold.

### 3.2 Molecular data

Thirty‐three mitochondrial *COI* sequences (9 from specimens morphologically identified as *R. quelen*, 11 as *R. voulezi* and 13 as *R. branneri*) of 566 bp in size were obtained in this study, in addition to six reference sequences from *R. quelen*, *R. voulezi* and *R. branneri* (Ribolli et al., 2017), with the following nucleotide frequencies: adenine (A) = 24.2%, thymine (T) = 29.7%, guanine (G) = 17.4% and cytosine (C) = 28.7%. The alignment of *COI* sequences revealed the existence of 533 conserved sites and 28 variable and Pi sites.

**FIGURE 5:**
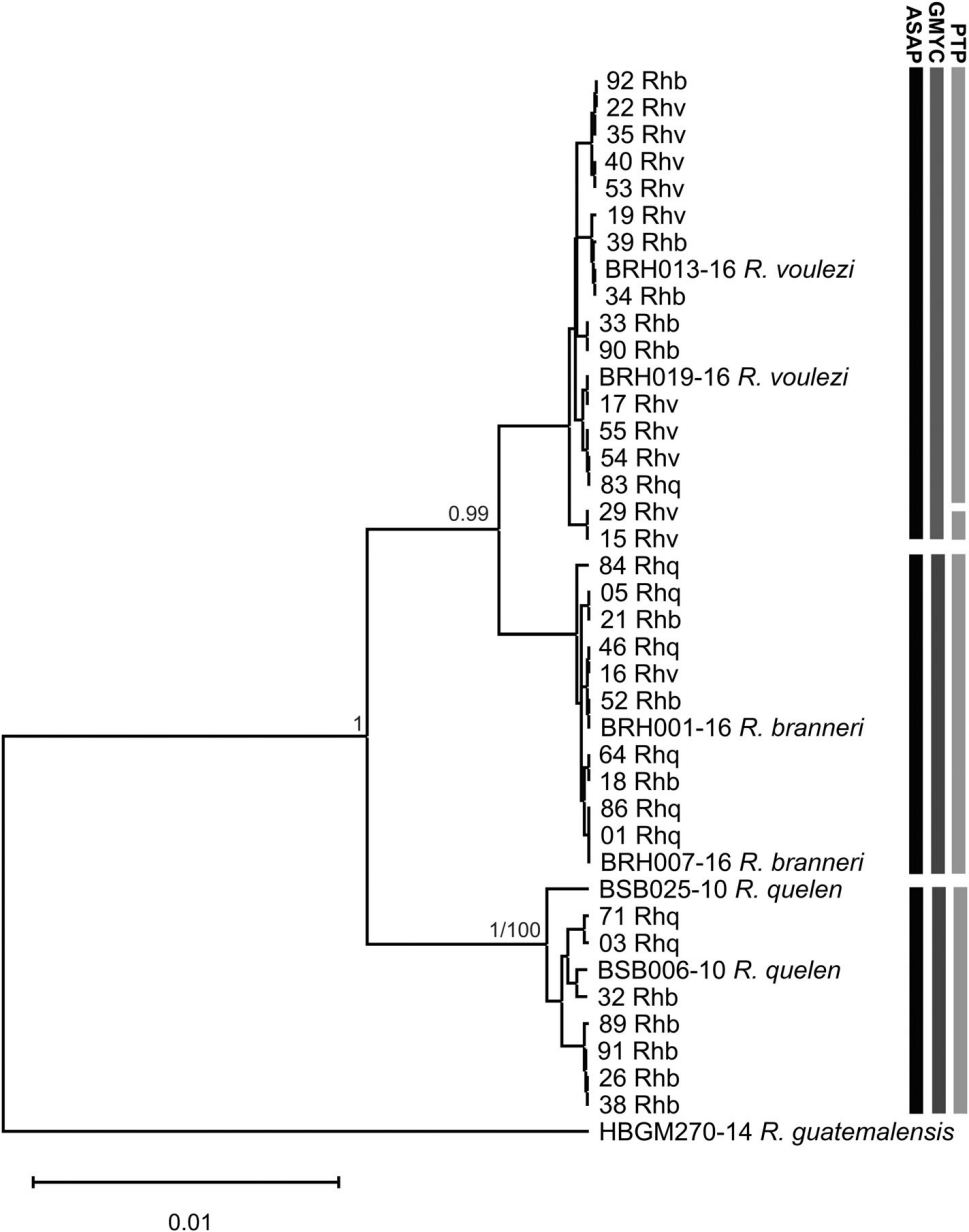
Bayesian tree constructed based on the nucleotide sequences of the mitochondrial gene cytochrome c oxidase I (*COI*) from specimens morphologically identified as *Rhamdia quelen* (Rhq) from the Paraná III River basin and *Rhamdia branneri* (Rhb) and *Rhamdia voulezi* (Rhv) from the lower Iguaçu River basin. Reference sequences, identified with the initials BRH and BSB, were obtained from the BOLD database and refer to the study by Ribolli et al. (2017).

The BA (Figure 5) and ML (Figure S1) dendrograms based on *COI* sequences revealed the formation of three distinct groups, each group corresponding to each of the three *Rhamdia* species, according to Ribolli et al. (2017). However, in our study, the molecular identification of about 60% of the samples did not coincide with the taxonomic identification. The clade referring to *R. voulezi* included specimens of both the sympatric species *R. branneri* and of *R. quelen*. The same was observed for the *R. branneri* clade, which grouped specimens morphologically identified as *R. voulezi* and *R. quelen*. In the *R. quelen* clade, specimens morphologically identified as *R. branneri* were also observed. In other words, samples identified as *R. branneri* and *R. voulezi*, which are endemic to the Iguaçu River basin, were grouped with *R. quelen* from the Paraná III basin and vice versa.

Clade formation was corroborated by species delimitation analyses (Figure 5), which identified three to four MOTUs, depending on the method, among *Rhamdia* specimens from the Paraná III and lower Iguaçu river basins. Both the GMYC species delimitation method, based on the ultrametric BA tree, and the ASAP method, based on genetic distances, identified three MOTUs, each MOTU referring to each of the three *Rhamdia* species, according to the reference sequences of Ribolli et al. (2017). The PTP delimitation method, based on the ML tree, recovered four MOTUs, two referring to *R. quelen* and two referring to the species of the lower Iguaçu (*R. branneri* and *R. voulezi*).

The mean values of interspecific (or inter‐MOTUs) genetic distance ranged from 1.47% to 4.48%, with the highest values observed between *R. quelen* and the congener species of the lower Iguaçu. Intraspecific (or intra‐MOTUs) genetic distances ranged from 0.03%, for both *R. voulezi* and *R. branneri*, to 0.57%, for *R. quelen*, with a mean value of 0.21 ± 0.09% (Table 2). Fourteen diagnostic *COI* nucleotides were identified for *R. quelen*, six for *R. branneri* and one for *R. voulezi* (Table 3). A greater number of mitochondrial haplotypes (six) were observed among the specimens belonging to the MOTU of *R. quelen*. In contrast, only two haplotypes were identified among the specimens of *R. voulezi* and two other haplotypes among the specimens of *R. branneri*.

**TABLE 2:** K2P genetic distances ± standard deviations, in percentage, intra‐ (in bold) and inter‐molecular operational taxonomic units (MOTUs), of *Rhamdia* populations from the Paraná III and lower Iguaçu river basins.

	*Rhamdia branneri*	*Rhamdia voulezi*	*Rhamdia quelen*
*R. branneri*	**0.03 ± 0.03**		
*R. voulezi*	1.47 ± 0.55	**0.03 ± 0.04**	
*R. quelen*	4.48 ± 0.81	3.37 ± 0.77	**0.57 ± 0.21**
*Rhamdia guatemalensis*	9.12 ± 1.34	8.27 ± 1.36	9.12 ± 1.40

*Note*: Values obtained are based on the nucleotide sequences of the mitochondrial gene cytochrome c oxidase I (*COI*) (barcodes DNA).

**TABLE 3:** Collection site, variable nucleotide sites, haplotypes of mitochondrial gene cytochrome c oxidase I (*COI*) molecular markers of *Rhamdia* specimens, and respective accession numbers.

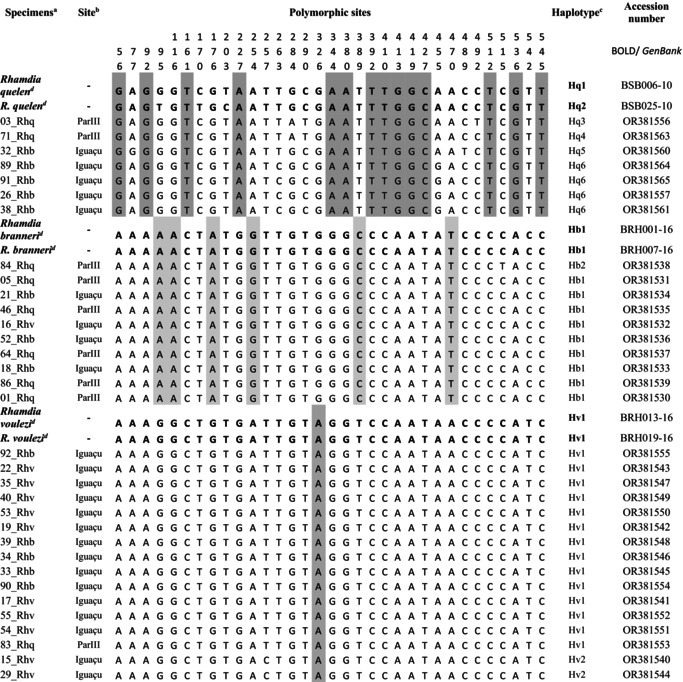

*Note*: Diagnostic sites are highlighted in grey. The samples are listed according to molecular identification using the reference sequences of Ribolli et al. (2017)

^a^ Sample codes according to the morphological identification of *R. quelen* (Rhq), *R. branneri* (Rhb) and *R. voulezi* (Rhv).

^b^ Paraná III (ParIII) and lower Iguaçu (Iguaçu) river basins.

^c^ Hq, mitochondrial haplotypes of *R. quelen*; Hb, mitochondrial haplotypes of *R. branneri*; Hv, mitochondrial haplotypes of *R. voulezi*.

^d^ Reference sequences.

## 4 DISCUSSION

The morphometric analyses indicated segregation of the populations based on the taxonomy and site of collection. Specimens collected along the Paraná III River basin, which were morphologically identified as *R. quelen*, formed a distinct group from those collected in the lower Iguaçu basin, morphologically identified as *R*. *branneri* or *R. voulezi*. Our results revealed differences among species in morphometric proportions, especially for adipose‐fin base length, dorsal‐fin spine length, caudal peduncle width, dorsal‐fin height, snout length, eye diameter, external mental barbel length and maxillary barbel length.

Similarly, DNA barcoding and species delimitation analyses identified *R. quelen*, *R. branneri* and *R. voulezi* as distinct MOTUs, corroborating the results of Ribolli et al. (2017) and the validity of the three *Rhamdia* species. However, the taxonomic and morphometric identification of most of the samples analysed here did not coincide with the molecular identification. This fact may be due to (i) incorrect morphological identification of the specimens and/or (ii) escapes of pure species and/or interspecific hybrids from fish farms.

The taxonomy of *Rhamdia* species is complex because they are morphologically similar; thus, an identification error is not uncommon (a fact that was also evidenced by Ribolli et al., 2021). Despite this, *R. quelen*, *R. branneri* and *R. voulezi* can be differentiated from each other by specialists. Mise et al. (2013) showed ecomorphological separation between the endemic species from Iguaçu, considering them ecologically distinct. Although *R. branneri* has characteristics related to benthic fish, *R. voulezi* presents aspects related to pelagic and lentic habitats (Mise et al., 2013). Garavello and Shibatta (2016) reported that *R. branneri* and *R. voulezi* (as well as the *R*. cf. *quelen* population of the Tibagi River basin) differ in dorsal‐fin and adipose‐fin morphology, structure and length of mentonian and maxillary barbels, pigmentation and morphometric characteristics. Moreover, the great endemism observed in the Iguaçu basin corroborates the distinction of its *Rhamdia* species (Baumgartner et al., 2012; Garavello & Shibatta, 2016).

Molecular analyses (DNA barcoding) also indicated differentiation among *R. quelen*, *R. branneri* and *R. voulezi*. Ribolli et al. (2017, 2021) confirmed the occurrence of *R. branneri* and *R. voulezi* in the Iguaçu River basin, with interspecific genetic distance values of about 1.4%. The differentiation is even greater between *R. quelen* and species from Iguaçu (3.2%–3.9%; Ribolli et al., 2017). Scaranto et al. (2018) also found evidence supporting the validity of the three species, with inter‐MOTUs genetic distance values ranging from 1.4% to 2.4% between *R. branneri* and *R. voulezi*, 3.2% to 3.3% between *R. quelen* and *R. voulezi* and 3.7% to 4.4% between *R. quelen* and *R. branneri*. Similar values were found among the *Rhamdia* species of the lower Iguaçu and Paraná III river basins analysed in this study, based on the clusters defined in the ML and BA dendrograms. The greater genetic differentiation observed between *R. quelen*, from the Paraná III River basin, and the lower Iguaçu populations (*R. branneri* and *R*. *voulezi*) is expected, as species from different river basins tend to be more genetically distinct from each other, mainly due to the allopatric speciation process (Lovejoy & Araújo, 2000).

Studies have used the barcoding cut‐off threshold of 2% for the species delimitation of Neotropical fish (Ward et al., 2009). However, using this 2% threshold, our molecular identification would be underestimated as the genetic distance between *R. branneri* and *R. voulezi* was 1.47%. Using the optimum threshold (OT) value of 0.77%, estimated specifically for *Rhamdia* species by Ribolli et al. (2017), as the limit between intra‐ and interspecific distances, the presence of the three species is supported. Also, 21 diagnostic nucleotides were identified in *Rhamdia* species, allowing the correct specific identification (or diagnosis) of a specimen based on the *COI* sequences (DNA barcoding) (Wong et al., 2009).

Given the well‐established differentiation among *R. quelen*, *R. branneri* and *R. voulezi* (Baumgartner et al., 2012; Garavello & Shibatta, 2016; Mise et al., 2013; Ribolli et al., 2017, 2021; Scaranto et al., 2018), the detection of mitochondrial haplotypes (*COI*) of *R. quelen* in the lower Iguaçu River basin or *R. branneri* and *R. voulezi* in the Paraná III basin is an important evidence of fish farm escapes of pure species or interspecific hybrids of *Rhamdia*. Determining the exact cause of these specimens in nature is difficult, but fish farm escapes seem to be the most likely cause, as aquaculture is considered the main source of introduction of exotic species into Neotropical reservoirs (Ortega et al., 2015). These illegal fish farm escapes are mainly attributable to the occupation of riverbanks and the lack of specific laws and regulations (Orsi & Agostinho, 1999).

The morphometric identification of specimens of *R. quelen*, *R. branneri* and *R. voulezi*, together with the molecular identification that did not correspond to their morphological species, for most samples, strengthens the hypothesis of occurrence of interspecific hybrid specimens, which have morphological and morphometric characteristics of one species but mitochondrial DNA of another *Rhamdia* species. The possible hybrids found here could not occur naturally, as *R. quelen* has distinct natural distribution of *R. branneri* and *R. voulezi*. Ribolli et al. (2021) also observed specimens morphologically identified as *R. quelen* grouped with species from the Iguaçu River (*R. branneri* and *R. voulezi*); however, they considered them probably misidentified.

The challenges of identifying and distinguishing different species that are morphologically similar and are used in aquaculture have led to the production and cultivation of these fishes as a single species (Hashimoto et al., 2014). *R. quelen*, in a broad sense, is a native fish of commercial importance in southern Brazil (Baldisserotto, 2009) and has shown great potential for fish farming due to its accelerated growth, cold resistance, feed efficiency, resistance to handling and tasty meat (Fracalossi et al., 2004). The ease of manipulating fish gametes in fish farms and the remarkable morphological similarity among *Rhamdia* species contribute to the occurrence of intentional or accidental interspecific breeding (Scaranto et al., 2018). Also, *R. branneri* and *R. voulezi* are often grown and marketed under the same common name ‘silver catfish’ (‘jundiá’), the same common name used for *R. quelen* (Ribolli et al., 2017). Thus, the risk of producing interspecific hybrids of *Rhamdia* is high.

Although the use of interspecific and intergeneric freshwater hybrids is usual for genetic improvement (Hashimoto et al., 2011), the production of hybrid catfish in Brazil is still in its early stages, and the most commonly used species in aquaculture belong to the Pimelodidae family (Hashimoto et al., 2016). Therefore, it is most likely that accidental interspecific crosses have been occurring among *Rhamdia* species. Interspecific hybrids represent potential ecological risks as competitors and may cause genetic introgression if they are viable and fertile hybrids, affecting wild populations and causing irreversible problems (Fitzpatrick et al., 2015). Yet, few legislations regulating fish breeding exist in Brazil (Hashimoto et al., 2013; Suplicy, 2007). The possibility of pure or hybrid cultivated specimens reaching the natural environment through escapes may cause introgression of the genetic material in natural stocks, posing a risk to the genetic integrity of native species (Prado et al., 2012; Scaranto et al., 2018). The evidence of genetic contamination in native populations has already been reported by Scaranto et al. (2018), with the occurrence of cultivated *R. branneri* specimens in natural environments. The genetic identification of fish is a powerful tool for conservation of fish species (Carvalho et al., 2008; Scaranto et al., 2018). As they are target species for fishing and are widely cultivated in southern Brazil, the correct delimitation of *Rhamdia* species is crucial, as it minimizes identification errors, avoids unintentional interspecific breeding in fish farms or repopulation projects and reduces the risk of contamination of natural stocks (Ribolli et al., 2021). Likewise, identifying interspecific hybrids of *Rhamdia*, both in natural and cultivation environments, is necessary. However, because mitochondrial DNA has predominantly maternal transmission, nuclear diagnostic markers could be employed to identify interspecific hybrids of *Rhamdia*. Therefore, future studies should focus on identifying nuclear markers capable of distinguishing and characterizing *R. quelen*, *R. branneri* and *R. voulezi*, as well as F1 and post‐F1 hybrids of *Rhamdia*.

## 5 CONCLUSIONS

Morphometric and molecular analyses efficiently differentiated *Rhamdia* species. The segregation of the specimens based on the morphometric data coincided with the taxonomy and site of occurrence of the species; however, the molecular identification (barcodes DNA) of most specimens did not coincide with the taxonomic identification. This may be due to incorrect morphological identification of the specimens, escapes of pure specimens from fish farms and/or the occurrence of interspecific hybrids of *Rhamdia*, also due to escapes. The results obtained in this study indicate that the latter hypothesis is the most likely. The occurrence of interspecific hybrids of *Rhamdia* in Neotropical rivers implies a serious threat to the genetic integrity of natural stocks and the conservation of species. Although the production of interspecific hybrids of freshwater fish is common in Brazil (Hashimoto et al., 2011), there are currently no laws regulating this practice (Hashimoto et al., 2013). The results obtained herein reinforce the need for stronger measures in fish farming facilities to prevent intentional or accidental escapes of cultivated specimens into natural ecosystems. Genetic tools should also be applied to monitor fish and fish hybrids in both wild and cultivation environments as a preventive measure to support the sustainable development of aquaculture.

## AUTHOR CONTRIBUTIONS

Thaís Souto Bignotto led all the molecular experiments, analysed the data, conceived and designed the study, and wrote the first draft of the manuscript; Herivelto Beck de Souza conducted the morphometric and molecular experiments; Rafael Clovis da Silva Bronzim conducted the molecular experiments; Thiago Cintra Maniglia assisted in the molecular experiments, provided laboratory facilities for molecular analysis and contributed to the design of the study; Dirceu Baumgartner led the morphometric analyses, analysed the data, conceived and designed the study. All authors read, revised and approved the final version of the manuscript.

## Supporting information


**Figure S1.** Maximum likelihood tree based on the mitochondrial marker cytochrome c oxidase subunit I (COI) of specimens morphologically identified as *Rhamdia quelen* (Rhq), *Rhamdia voulezi* (Rhv) and *Rhamdia branneri* (Rhb) from the Paraná III and lower Iguaçu River basins.
