# Supplementary figures and images for: Integrative morphometric and molecular analyses reveal possible genetic contamination of silver catfish populations of the genus Rhamdia in Neotropical River basins
Source: J Fish Biol. 2025 Apr 16;107(2):535–46. doi: 10.1111/jfb.70057 (PMC12360138; doi:10.1111/jfb.70057)

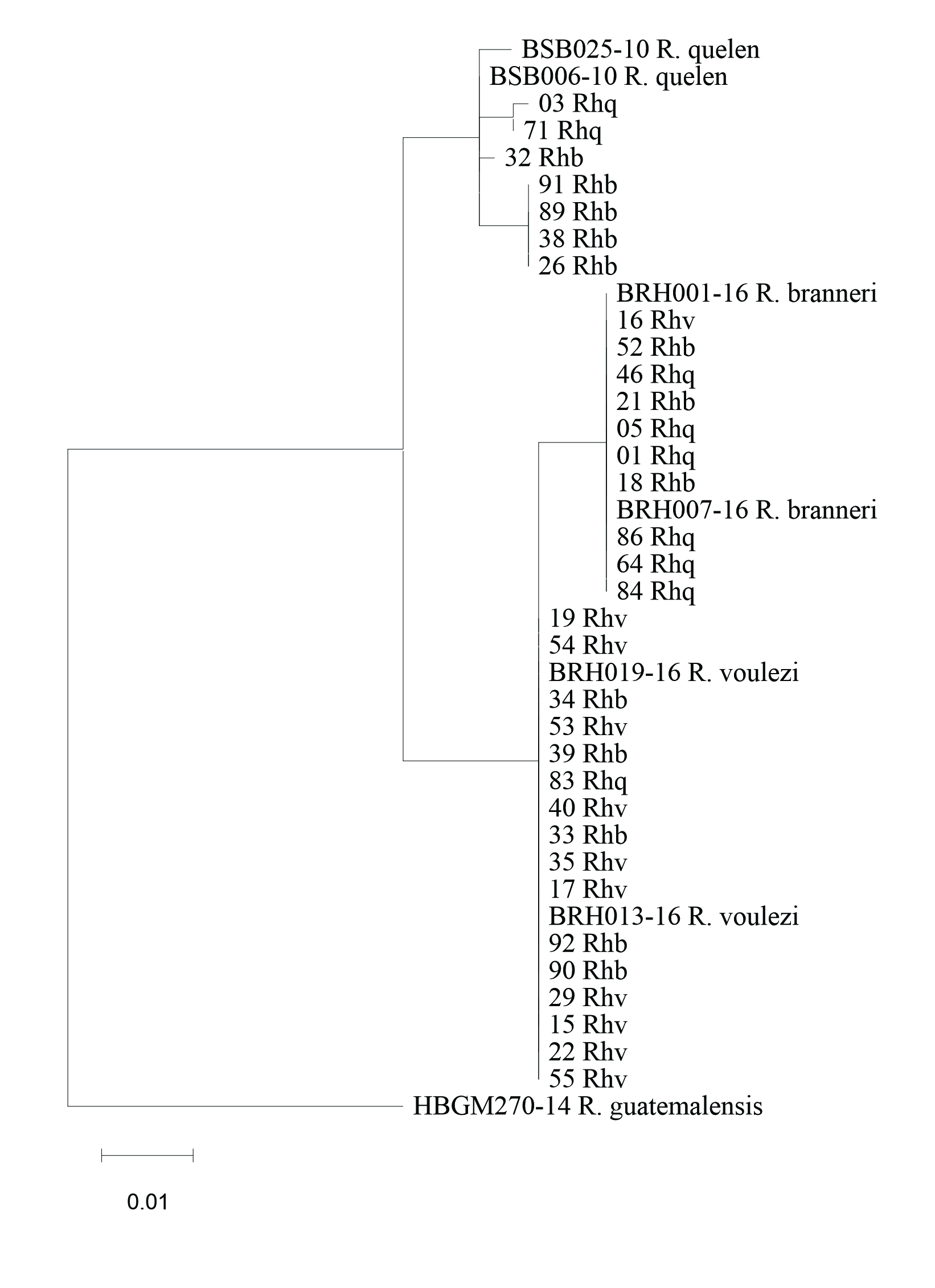

Supplement: Supplementary file 1 — Figure S1. Maximum likelihood tree based on the mitochondrial marker cytochrome c oxidase subunit I (COI) of specimens morphologically identified as Rhamdia quelen (Rhq), Rhamdia voulezi (Rhv) and Rhamdia branneri (Rhb) from the Paraná III and lower Iguaçu River basins. [file JFB-107-535-s001.tif]
